# Myocardial Revascularization for the Elderly: Current Options, Role of Off-pump Coronary Artery Bypass Grafting and Outcomes

**DOI:** 10.2174/157340312801215809

**Published:** 2012-02

**Authors:** Shahzad G Raja

**Affiliations:** Department of Cardiac Surgery, Harefield Hospital, Hill End Road, Harefield, UB9 6JH, United Kingdom

**Keywords:** Cardiopulmonary bypass, coronary artery bypass grafting, elderly, octogenarians, off-pump coronary artery bypass.

## Abstract

The increase in life expectancy has confronted cardiac surgery with a rapidly growing population of elderly patients requiring surgical myocardial revascularization. Recent advances in surgical and anesthetic techniques and improvements in postoperative care have made coronary artery bypass grafting an established therapeutic option for the treatment of coronary artery disease in this group of patients. However, conventional coronary artery bypass grafting on cardiopulmonary bypass is associated with significant risk and related morbidity and mortality in the elderly. In recent years off-pump coronary artery bypass grafting has emerged as a safe and less invasive strategy for surgical myocardial revascularization. Off-pump coronary artery bypass grafting by avoiding the deleterious effects of cardiopulmonary bypass can offer potential benefits to elderly patients requiring surgical myocardial revascularization. This review article provides an overview of the age-related cardiovascular changes, epidemiology of coronary artery disease in the elderly and focuses on outcomes of surgical myocardial revascularization with special emphasis on the impact of off-pump coro-nary artery bypass surgery in the elderly.

## INTRODUCTION

The elderly population in the Western world is growing exponentially. The age group defined as “elderly” in the literature has gradually increased from ≥65 years to ≥80 years. The greatest increase in numbers undergoing heart surgery has occurred in the oldest group of persons, those aged ≥85 years [1]. Among the 25 000 coronary artery bypass grafting (CABG) operations performed every year in the United Kingdom, almost a quarter of the patients are aged >70 years, and 8% are >75 years [2]. This aging surgical population, not unexpectedly, has a relatively greater prevalence of cerebrovascular disease, left ventricular dysfunction, diabetes mellitus, chronic obstructive pulmonary disease, renal impairment and peripheral arterial disease [3-5]. Therefore, such elderly people with multiple comorbidities tend to have a high rate of complications after CABG [3]. Indeed, old age alone (ie, >75 years) is an independent risk factor for poor outcomes after CABG [4].

## AGE-RELATED CARDIOVASCULAR CHANGES

In all, 80% of patients >80 years have identifiable cardiovascular disease [6]. Age-related changes occur in small and large vessels and in the heart itself, reducing physiological reserves. Most patients show no signs of impaired hemodynamic performance at baseline, but stresses of anesthesia will often uncover their limited cardiac reserve [6].

### Heart

Aging is associated with numerous molecular, ionic, biophysical and biochemical changes in the heart [5,7,8]. These changes affect protein function, mitochondrial oxidative phosphorylation, calcium kinetics, excitation-contraction coupling, myofilament activation, contractile response, matrix composition and regeneration, cell growth and size, and apoptosis [5].

Age-related changes in cardiac morphology are mostly the result of alterations of intracellular molecular and biochemical pathways. In turn, many of the changes in cardiac function with advancing age develop in response to underlying alterations in morphology. Ultimately, cardiac aging results in decreased mechanical and contractile efficiency, prolongation of the relaxation phase, stiffening of myocardial cells, mural connective tissue and valves, decreased number of myocytes, increased myocyte size, increased rate of myocyte apoptosis and blunted β-adrenoceptor-mediated contractile and inotropic response [9-11].

### Vasculature

Aging affects various aspects of vascular morphology and function. The large arteries dilate, their walls thicken, the wall matrix changes, elastolytic and collagenolytic activity increases and smooth muscle tone increases. As a result, vascular stiffness increases with advancing age [8,12,13]. Increased vascular stiffness leads to elevated systolic arterial pressure and pulse-wave velocity, and to early reflected pulse pressure waves and late peak systolic pressure, thereby augmenting aortic impedance and cardiac mechanical load [8]. In this way, arterial stiffening triggers a variety of cardiac adjustments. Some of these adjustments are additional and are similar to the age-related intrinsic changes in cardiac morphology and may, therefore, be expected to worsen cardiac performance [13]. 

### Coronary Circulation

Aging is associated with structural and functional changes in the coronary vasculature, which could affect myocardial perfusion with advancing age. The gradual age-related decline in coronary flow reserve may be a result of elevated baseline cardiac work and myocardial blood flow [14] or abnormal vasodilator capacity. Such a reduced dilator reserve may be the result of impaired endothelium-dependent dilation of large epicardial and resistance coronary vessels [15], decreased basal and stimulated release of nitric oxide by the coronary endothelium [16], or increased coronary vasoconstrictor effect of endothelin-1 (ET-1) [17]. Endothelin plasma concentrations increase with increasing age [18].****

## AGE AND ISCHEMIC HEART DISEASE

Aging affects cardiovascular risk factors, incidence and clinical manifestation of cardiac disease, treatment strategies and prognosis [13]. Cardiovascular disease is superimposed on age-associated changes in cardiac and vascular characteristics. The final pathophysiological mechanism and clinical presentation result from an interaction between age-related changes in cardiovascular physiology and cardiovascular disease.

### Chronic Ischemic Heart Disease

The diagnosis of ischemic heart disease may be more difficult in the elderly. Reduced physical activity with age limits the occurrence of demand angina. Possibly related to the age-related changes in myocardial compliance and diastolic relaxation, dyspnea, rather than pain, may dominate the clinical picture of myocardial ischemia and infarction. The predictive value of a negative exercise stress test is low in a population with a high prevalence of ischemic heart disease. As many elderly people are unable to exercise to 85–90% of their predicted maximum heart rate, a pharmacological stress test with thallium scan or echocardiogram is often of greater diagnostic accuracy [13].

Although the goal and choice of anti-ischemic treatment are generally similar in young and old patients, the elderly must be expected to be more sensitive to the hypotensive effects of certain anti-ischemic drugs because of blunted baroreceptor reflex activity and sympathetic responsiveness, and increased myocardial and vascular stiffness. As a result of the same age-related decrease in sympathetic responsiveness, myocardial ischemia is less likely to be provoked by adrenergic-mediated increases in myocardial oxygen demand and the benefit derived from treatment with β-adrenoceptor blockers may be reduced.

### Acute Myocardial Infarction

In the elderly, in-hospital and subsequent mortality, reinfarction and complications are all increased [19]. Likewise, perioperative myocardial infarction carries a considerably higher mortality in the elderly [13,20]. In addition, age is an independent predictor of adverse outcome following various therapeutic interventions, such as percutaneous transluminal angioplasty, coronary stenting and thrombolysis [13,21].

Increased mortality and morbidity in the elderly after myocardial infarction and therapeutic cardiovascular interventions have many causes. They include greater impairment of baseline left ventricular function, more advanced multivessel disease, higher rate of major complications [21], greater risk of cardiac rupture despite comparable infarct size (probably on the basis of age-related morphological changes), higher rate of vascular complications [21], increased incidence of non-cardiac complications (e.g. stroke and hemorrhage), greater likelihood of interventions being performed under emergency conditions [21], lower procedural success [21], more contraindications to thrombolytic therapy, longer times between onset of symptoms and presentation for evaluation and treatment, blunted catecholamine response, impaired renal and respiratory reserve, and coexisting diseases [13, 21,22]. 

## DEMOGRAPHICS OF ISCHEMIC HEART DISEASE IN THE ELDERLY

Expanded life span among the elderly population in both North America and western European countries has increased the prevalence of coronary artery disease. The population of the 85 years and older increases 10 times faster than the average population. Average life expectancy at 80 years exceeds 8 years and is still around 6 years at 89 years [23]. According to the US statistics, up to 25% of octogenarians are significantly affected by cardiovascular disease [23]. Over the next 20 years, the absolute number of American citizens reaching at least 85 years is expected to double passing from 9.3 millions to 19.5 millions, which will make the demand for cardiovascular medical care literally explode [24].

Age is the most powerful predictor of cardiovascular adverse events after acute coronary artery syndrome [25]. For each aging decade, in-hospital death rises by 70%. Although citizens of at least 75 years old represent only 6% of the US population, they are responsible for 60% of the in-hospital deaths related to myocardial infarction (MI). According to the World Health Organization (WHO), prevalence of cardiovascular disease will increase by 120% in men and by 137% in women during the next two decades mainly due to the aging of the current population [23]. Although the average age of the first MI is 65.8 years in men and 70 years in women, candidates from both sexes are expected to survive this event by more than 15 years [25]. In spite of this astonishing demographic outbreak and the surge for medical care it will create, the elderly population still does not receive all the proper attention they deserve [26,27].

## CORONARY REVASCULARIZATION IN THE ELDERLY

Selection of the most appropriate treatment pathway for elderly patients with ischemic heart disease requires careful evaluation of the risks and benefits of three forms of treatment: medical therapy, percutaneous coronary intervention (PCI) and CABG surgery, on an individual basis. Two recent studies have examined outcomes in the elderly when assigned to revascularization versus optimal medical therapy [28,29]. Trial of invasive versus medical therapy in elderly patients with chronic symptomatic coronary artery disease (TIME), a prospective randomized trial involving 305 patients with angina, found that, at 6 months revascularization, percutaneous or surgical, was associated with an improved quality of light and fewer major cardiac events (19 vs 49%) than optimized medical treatment [28]. The authors concluded that elderly patients with symptoms not controlled by medication benefit from angiography with a view to revascularization. The Alberta Provincial PRoject for Outcome Assessment in Coronary Heart disease (APPROACH) observational study analyzed the absolute risk reduction in late mortality following CABG or PCI compared with medical therapy, based on a cardiac catheterization database of more than 6000 elderly patients with ischemic heart disease [29]. Intervention improved outcome in all groups compared with pharmacological therapy and persisted after accounting for potential selection biases; paradoxically, the greatest benefit was found in the oldest patients (>80 years of age), with survival improvements of 17.0% for CABG and 11.3% for PCI, respectively.

Recently, to appreciate how modern therapeutics could benefit the elderly with acute coronary syndrome, the CRUSADE (Can Rapid Risk Stratification of Unstable Angina Patients Suppress Adverse Outcomes with Early Implementation of the ACC/AHA Outcomes Guidelines) investigators revised the results of the study, including age/stratification designed to compare contemporary in-hospital treatment patterns and outcomes of elderly patients with non-ST-segment elevation acute coronary syndrome (ACS) with their younger counterparts [30]. Patients were clustered into four age groups: below 65, 65–74, 75–84, and above 85 years old. In-hospital death increased with age from 1.9% below 65 years to 11.5% above 85 years, even after adjustment for risk factors (odds ratio 3.00). Occurrence of other in-hospital events correlated with age, cardiac failure, stroke, recurrent MI, and transfusion need being the most common. Even though close to 60% of the 85 years and older patients had an in-hospital coronary angiogram, less than 20% had PCI performed compared with 50% in those below 65 years of age. On average, CABG was performed in 10% of the patients until the age of 80 years abruptly dwindling down after. Age correlated with a lesser use of aspirin, beta-blockers, heparin, clopidogrel, and platelet glycoprotein IIb/IIIa inhibitors, even though the use of these five guidelines-recommended therapies was associated with lower in-hospital mortality. This remained true even after correcting for other risk factors regardless of patient age. Authors concluded that future work was required to clarify treatment and prevention of ACS in the elderly [30]. 

## OUTCOMES OF PCI IN THE ELDERLY

The results of both PCI and CABG are improving [31,32], although the exponential rise of PCI over the last decade has made it the principal method of coronary revascularization. In the elderly, PCI is beneficial, but the rate of complications increases disproportionately [32,33] and coronary anatomy is often less amenable [34]. A study of PCI in 8828 octogenarians during 1998–2000 found angiographic success in 93% with stents placed in 75% of patients [35]. Post-procedural mean length of stay was 3.3 days with an in-hospital mortality of 3.77%, decreasing to 1.35% in patients without recent MI (p < 0.0001).

Over the past decade, device technology of drug-eluting stents has evolved with a concomitant improvement in outcomes of PCI in the elderly. A recently published report from the randomized multicentre SPIRIT III trial comparing the outcomes in elderly and younger patients treated with everolimus-eluting stent (EES) versus paclitaxel-eluting stent (PES) has shown that implantation of both EES and PES appears to be safe in elderly patients [36]. However, currently published experience of the use of drug-eluting stents in the elderly is limited. Furthermore, the long-term outcomes of current PCI technology and the clinical relevance of incomplete revascularization following PCI in the elderly are yet to be determined.

## OUTCOMES OF CONVENTIONAL CORONARY ARTERY BYPASS GRAFTING IN THE ELDERLY

Although old age is seen as a significant risk factor for conventional cardiac surgery, recent publications have reported favorable outcome in elderly patients. CABG in octogenarians relieves angina effectively [2,37]. The overall mortality after CABG in elderly patients has steadily declined over the years with improvements in surgical techniques. The crude survival rates after CABG in individuals aged >75 years in the UK has increased from 92.4% (n = 821) in 1998 to 94.1% (n = 1804) in 2001 [2]. In 2008, patients > 80 years made up 4.4% of all CABG operations performed in the UK with a marked fall in the mortality of patients over the age of 75 years from 5.0% in 2004 to 3.4% in 2008 according to the sixth national adult cardiac surgery database report of the Society for Cardiothoracic Surgery in Great Britain & Ireland. 

Modern literature acknowledges an operative mortality between 2.7 and 6.4% for isolated CABG and a 5-year life expectancy of 65% [38-40]. A large study found a 34% reduction in risk-adjusted operative mortality in elderly people (1982–96), apart from confirming a time-related increase in the prevalence of older patients and an increase in the preoperative risk profile of these patients. But, when compared with patients of a younger age group, those aged >75 years continue to have poorer short-term outcomes [1]. In an analysis of 6057 patients who underwent isolated CABG between 1996 and 2002, the 30-day mortality rate and the incidence of postoperative complications was found to largely escalate with age [41]. In-hospital outcomes and cost were examined among 2272 elderly people (≥75 years) and 9745 younger patients (<75 years) who underwent CABG between 1997 and 2001 in another study [42]. After controlling for clinical differences, age ≥75 years was found to be associated with a longer length of hospital stay, higher mortality rates and higher in-hospital cost [42]. 

Recent data from prospective and randomized trials have even reported more favorable outcome with conventional CABG surgery over PCI, especially for diabetes and patients above 65 years of age [40,43]. Over time, prevalence of postoperative complications and use of hospital resource have significantly declined in this population [39]. Maganti *et al*. [39] have reported a regression in operative mortality among octogenarians from 7.1 to 3.2% along with a decreased incidence of postoperative complications such as stroke, low cardiac output, and use of intra-aortic balloon pump. Concomitantly, they documented an increased prevalence of risk factors such as diabetes, dyslipidemia, hypertension, and left main disease in this population.

As CABG effectively relieves angina and may prolong survival, improved quality of life could be expected after surgery. Equally important to determining the overall quality of life is the need to determine the effect that CABG has on physical and mental health. Data available to help clinicians identify those elderly patients who are likely to have an improvement in quality of life after CABG are limited. Although hospitalisation may be longer for elderly patients, physiological, psychological and social recovery patterns through the first 6 weeks postoperatively have been reported to be similar to those of a younger age group [44]. In a study which used self-reported health questionnaires in 1744 patients aged >65 years undergoing CABG, significant improvements were noted in quality of life after a 6-month follow-up [45]. This benefit was present across all age groups and was found to be particularly magnified in patients who had a poorer preoperative health status. Hedeshian *et al*. [37] found that patients aged >70 years presented for CABG at a lower functional level than younger patients, but the marked improvement in functional capacity that occurred after surgery was comparable among all age groups. More recently, survivors among octogenarians who underwent isolated CABG were found to have an excellent quality of life for up to 5 years after surgery [45]. Hence, excellent long-term survival after CABG in elderly people may indeed be accompanied by an equally satisfactory quality of life in the majority.

## RATIONALE FOR THE USE OF OFF-PUMP CORONARY ARTERY BYPASS GRAFTING IN THE ELDERLY

Off-pump coronary artery bypass (OPCAB) grafting, in recent years, has emerged as an effective surgical technique, and may be of particular benefit in high-risk populations including the elderly. Conventional CABG using cardiopulmonary bypass (CPB) and cardioplegic arrest has, for many years, represented the “gold standard” in coronary revascularisation. However, over the past decade OPCAB has gained increasing popularity. Resurgence of interest in OPCAB is associated with the expectation that avoiding deleterious effects of the CPB pump leads to better outcomes and possibly decreased costs and resource utilization [46]. OPCAB is clearly associated with blunting of systemic inflammatory response syndrome (SIRS) [47]. SIRS results from a cascade of events generated by the contact of plasma proteases and blood cells with the gaseous interface and bioincompatible surfaces of the CPB machine. High-risk patients including the elderly are particularly susceptible to damage by these inflammatory mediators [48,49]. There is overwhelming evidence from randomized controlled trials, propensity score analyses and observational studies that OPCAB is associated with reductions in the risks for stroke, atrial fibrillation, wound infection, and acute kidney injury [50-56]. OPCAB also reduces transfusion and inotrope requirements, ventilation time, intensive care unit and hospital stays, and in-hospital and 1-year direct costs [50-56]. Since atrial fibrillation, decline of neurocognitive functions, delirium, stroke, increased length of stay, and renal failure are common complications more frequently encountered in this population [57,58], it makes sense to offer OPCAB surgery to the elderly patients. 

## OUTCOMES OF OFF-PUMP CORONARY ARTERY BYPASS GRAFTING IN THE ELDERLY

Advanced age is an independent risk factor for mortality after CABG surgery [57]. Avoiding CPB can hamper complications and mortality related to revascularization surgery [59-62]. Elderly patients are particularly sensitive to the side effects of the CPB and require increased resource utilization compared with younger patients [63-65]. The current literature comparing on and off-pump technique in elderly patients is quite modest and reflects the difficulty to enroll these very fragile patients in prospective randomized studies. Nevertheless, many nonrandomized studies and subgroup analyses of randomized trials, have demonstrated potential benefit of avoiding CPB in the elderly population (Tables **1** and **2**) [66-97].

### Mortality

Age is an independent predictor of postoperative morbidity and mortality among elderly patients undergoing CABG [64,98]. In such patients minimizing the invasiveness of the surgical procedure by avoiding the use of CPB is an attractive option to improve outcomes.

Panesar *et al*. [65] in an extensive review of the 1999-2005 literature targeting older patients, operated either on or off-pump, have come up with an exhaustive meta-analysis of the topic. They identified 1533 patients in the OPCAB group and 3388 patients in conventional group. Primary endpoint was operative mortality and stroke, and secondary endpoints were prevalence of atrial fibrillation, renal failure, and length of hospital stay. They categorized the patients into three distinct groups: above 70 years, above 75 years, and above 80 years old. At large, operative mortality was diminished in the OPCAB group (*p* = 0.01) and more specifically in the oldest group (*p *= 0.007).

Majority of the publications since 2005, analyzing impact of OPCAB and CPB on mortality in elderly, report similar in-hospital mortality rates for conventional CABG and OPCAB with the overall early mortality for this high-risk group ranging from 4.6% to 5.9% (Table **2**) [66,67,69-71]. However, some recent nonrandomized studies such as the propensity score analysis by Meco *et al*. [72] have shown that mortality is higher in the patient group operated with CPB as compared to patients operated without CPB (12.2% vs 1.3%, *p* = 0.01). Furthermore, their logistic regression analysis has shown that avoiding CPB is an independent protective factor for mortality and morbidity in the elderly. 

### Stroke

Coronary artery bypass surgery is associated with adverse neurological complications, of which stroke is the most debilitating. Despite the improvement in surgical techniques and cardioplegic agents, along with the introduction of membrane oxygenators and in-line filtration, there is a persistent stroke rate associated with CABG ranging from 1% to 5% [99,100]. It is estimated that 3000 to 15,000 patients each year suffer a stroke in the perioperative period after CABG [99,100]. Age is identified as an independent predictor of stroke [101,102]. The increasing risk with older age has been related to the higher prevalence of diseased aorta which may lead to perioperative atheroembolism from aortic arch plaque [103]. Indeed, the identification of an atherosclerotic ascending aorta has been reported as the single, most significant marker for an adverse cerebral outcome after CABG [103], reflecting the role of aortic atheroembolism as the main cause of ischemic stroke [104].

Over the last decade, prevalence of stroke after CABG surgery has significantly decreased in octogenarians, and recent reports quote a risk of 2% (Table **2**) [39]. Prevalence still remained twice as high as what is currently seen in younger patients [105]. Prevention definitely remains the key issue in the management of this complication [23]. Athanasiou *et al*. [62] in their meta-analysis of all observational studies, published in MEDLINE between 1999 and 2002, showed that the OPCAB technique was associated with significantly lower incidence of stroke in elderly patients compared with the CPB technique (1% vs 3%), with an odds ratio of 0.38% to 95% (confidence interval [CI], 0.22 to 0.65). Similar results have been reported by Panesar *et al*. [65] Their meta-analysis showed a significant reduction in OPCAB patients above 70 years old compared with conventional CABG surgery (0.9% versus 3.9%, p = 0.05). The strongest benefit was observed in the octogenarians. Even though history of previous stroke was more frequent in OPCAB surgery octogenarians patients (15.4 versus 8.9%), occurrence of new postoperative stroke was significantly higher with conventional surgery (p = 0.002). Less invasive nature of OPCAB with minimal manipulation of atheromatous aorta could account for the reduction of postoperative stroke in the elderly.

### Cognitive Impairment

Cognitive deficits have been documented in a majority of patients after cardiac surgery [106]. Off-pump surgery has been shown to better preserve neurocognitive impairment than conventional CABG surgery [59,107-109]. The pathophysiology of these neurocognitive deficits appears to be multifactorial [110]. Prospective randomized trials specifically designed to explore the potential benefit of OPCAB surgery on postoperative neurocognitive functions have confirmed the benefit of OPCAB surgery in the first 3 months after surgery [59,70,74,107-109,111,112]. Interestingly, Jensen *et al*. [74] failed to show a difference in the incidence of cognitive dysfunction 3 months after either OPCAB or conventional CABG in a substudy of the randomized Best Bypass Surgery trial. In this study a total of 120 elderly patients (mean age 76 ± 4.5 years) underwent psychometric testing before surgery and at a mean of 103 ± 15 days postoperatively with a neuropsychological test battery that included 7 parameters from 4 tests. Cognitive dysfunction was defined as the occurrence of at least 2 of the 7 possible deficits. Secondary analysis was performed on the basis of the definition of a 20% decline in cognitive scores compared with baseline, and with z score analysis. Cognitive dysfunction was identified in 4 of the 54 patients (7.4%, 95% CI 2.1% to 17.9%) in the OPCAB group and 5 of the 51 patients (9.8%, 95% CI 3.3% to 21.4%) in the conventional CABG group. At 1 year follow-up the findings remained the same [70].

The reasons for the limited differences in cognitive outcome between the treatment groups observed in the study Jensen *et al*. [74] may be explained in several ways. When one examines the literature, the crucial step of finding a significant neurocognitive deficit is in determining the definition itself. The definition of a significant deficit varies, and the lower the threshold of “deficit” is determined to be, the more patients there will be who have a deficit. This level is arbitrary from research group to research group and varies from a deterioration of 1 SD in 1 or more tests, a deterioration of 20% or 25% in at least 1 or 2 tests, to the use of a standardized *z* score or composite *z* score [113]. The definition of cognitive dysfunction in the study by Jensen *et al*. [74] was more restrictive than the “20% criterion” and the definition with the *z* score. In the analyses of the test results from Jensen’s study [74], the evaluation of cognitive function was based on differences between preoperative and postoperative performance. Therefore, the association between early and late cognitive outcome could be explained by regression toward the mean [114], because generally, the use of scores favors patients with poor preoperative performance because of the “protective” effect of low baseline performance [113]. 

Another explanation involves the short-term follow-up in Jensen’s study [74], because it has been suggested that improved cognitive outcome with an OPCAB procedure may only become clear in the long term. van Dijk *et al*. [111] found an increasing incidence of cognitive decline from 3 to 12 months, and Newman *et al*. [115] found cognitive decline in 24% of patients 6 months after conventional CABG, which increased to 42% after 5 years. 

A final explanation might be that the OPCAB technique is a new source of cognitive dysfunction caused by decreased cerebral perfusion pressure during episodes of elevated central venous pressure and corresponding decreased arterial blood pressure, in connection with dislocation of the heart during surgical exposure of the posterior cardiac wall [116]. The influence of systemic mean arterial pressure during CPB and neurological outcome has been the subject of considerable debate. Commonly, a mean arterial pressure of 50 to 60 mm Hg when the patient is undergoing CPB is regarded as safe to avoid neurological complications [74]. 

### Postoperative Atrial Fibrillation

Atrial fibrillation (AF) is the most common complication after cardiac surgery, with little change over the past 2 decades [117]. Postoperative AF increases hospital morbidity and mortality and has significant adverse effects on patient recovery. The lengthening of hospital stay and the consequent burden caused by the occurrence of postoperative AF has led to an extensive scrutiny of predisposing factor and preventive strategies [118,119].

Aging increases the prevalence of AF by 44% in every 10 years [23]. At least three recent meta-analyses have questioned the influence of OPCAB surgery on postoperative AF. The first one included all observational studies comparing on and off-pump revascularization on patients above 70 years of age and demonstrated a reduced incidence of postoperative AF in favor of OPCAB patients (21.9% versus 28.5%, p = 0.003) [105]. The second one included all randomized and propensity score-matched nonrandomized studies published between 2001 and 2003 comparing both techniques regardless of age [120]. Their results suggested that OPCAB surgery could reduce the incidence of postoperative AF in the general population (age <70 years), but the optimal ‘protective effect’ was not as strong as recorded in the older population. The meta-analysis on benefit of OPCAB in elderly by Panesar *et al*. [65] although targeting mostly in-hospital mortality and stroke, showed a benefit in favor of OPCAB for all groups above 70 years old confirming the findings of the other studies.

### Postoperative Renal Dysfunction

Renal dysfunction is a serious complication after coronary artery bypass surgery with cardiopulmonary bypass. Cardiopulmonary bypass-related non-pulsatile flow, hypothermia, hemolysis, systemic inflammatory reactions and emboli are mentioned as possible causes for this postoperative renal dysfunction [54]. In addition advanced age is an important predictor of postoperative renal dysfunction after CABG [121]. A recent propensity-based study conducted on 2041 patients confirmed these findings [122]. However, a meta-analysis of 6 randomized controlled trials and 16 observational studies, comprising 27,806 patients, failed to show strong benefit in the elderly population regarding OPCAB and renal failure [123]. Currently, the evidence on this issue is limited and further studies are required to clarify this topic.

### Resource Utilization

Advanced age is associated with diminished physiologic reserve and increased comorbid illnesses, including diabetes, chronic obstructive pulmonary disease, cerebrovascular disease, and peripheral vascular disease. These comorbidities lead to increased postoperative complications and resource utilization in this population. In an era of fiscal constraint and fixed resources, management of the elderly CABG patient poses significant medical and ethical challenges for both healthcare providers and healthcare administrators. Boyd *et al*. [97] have examined the cost of OPCAB and conventional CABG surgery in the geriatric population. They hypothesized that postoperative morbidity was responsible for longer intensive care unit (ICU) and hospital stays. They grouped together patients who had an ICU stay longer than 24 hours and/or a hospital stay longer than 8 days, and defined these individuals as adverse economic outcomes. The prevalence of adverse economic outcomes was four times higher in the CABG population (OPCAB 10% vs CABG 40%, *p* < 0.005). Furthermore, the reduction in hospital resources in the OPCAB patients resulted in an average savings of CAN $1,082 per patient in this group. These savings were based solely on decreases in hospital stay and ICU stay, and did not include additional savings yielded from decreased operating room equipment costs or blood product usage. Again the evidence on this topic is scant at present.

### Quality of Life

The success of cardiac surgery is not solely judged by its effects on mortality but also by its neuropsychological and emotional consequences, and by its influence on health-related quality of life (HRQoL) [124]. Changes in cognitive function have been associated with reduced HRQoL 1 year [124] and 5 years after cardiac surgery in terms of lower general health and a less productive working status [125]. A randomized study, using a post-test only design, compared medical treatment to invasive treatment in 113 patients with inducible ischemic heart disease. At an average follow-up of 36 months, more invasively treated patients had concentration difficulties but better HRQoL scores in the physical variables [126]. CABG appears to have a beneficial effect on psychological function and HRQoL for the majority of patients [127]. 

Jensen *et al*. [73] have conducted to date the first and only randomized study to evaluate the effect of off-pump versus on-pump CABG on changes in various aspects of HRQoL in elderly moderate to high-risk patients during the first 3 months after the operation. After randomization and before heart surgery, 120 consecutive patients were asked to fill in the Medical Outcomes Study Short Form 36 (SF-36) and Major Depression Inventory diagnostic scale for self-report of HRQoL. Three months after surgery, the same questionnaires were mailed to the patients. The response rate was 96.5%. At baseline, the groups were comparable except for a difference in educational level. Both groups improved in all eight SF-36 domains from baseline to 3 months. No statistical differences were seen between the groups except for changes in mean difference of role limitation due to emotional problems, which was significantly (*p* = .04) improved in favor of the on-pump group. Depression scores remained unchanged within and between the two surgical groups. Both on-pump and off-pump patients improved in health-related quality of life scores after CABG surgery. No clinically relevant difference between the groups could be demonstrated. However, despite being a randomized study, generalizations of these findings is limited by the fact that it is a single centre experience and there is need for further studies to verify whether or not there is an improvement in HRQoL among OPCAB elderly patients compared to patients undergoing on-pump CABG.

## COMMENT

Cardiac surgery has been increasingly offered to the elderly patients particularly octogenarians over the past decade. Increasing age is a well-established independent predictor of postoperative morbidity and mortality among patients undergoing CABG. In such patients minimizing the invasiveness of the surgical procedure by avoiding the use of CPB seems a reasonable strategy to improve outcomes. OPCAB due to its less invasive nature appears an attractive option to reduce the mortality and morbidity in high-risk elderly patients.

At present, the evidence comparing impact of OPCAB on postoperative complications in elderly fails to show a convincing benefit of avoiding CPB. At the author’s institution (Table **3**), with a significant bias for OPCAB revascularization in the elderly particularly octogenarians, outcomes of OPCAB are comparable to outcomes for CABG on CPB. In our experience the only benefits associated with OPCAB, were a shorter postoperative period of intensive therapy unit (ITU) stay and hospital stay along with lesser use of inotropes, blood product usage and re-exploration for bleeding Such findings although seemingly trivial, may translate into substantial savings in this group of patients with a higher-level of resource utilization perioperatively. 

It is however, important to mention that despite a shorter ITU and hospital stay among OPCAB patients lengthy rehabilitation was needed in elderly patients postoperatively irrespective of surgical strategy utilized. 

The current evidence on the topic is fraught with limitations. Majority of the studies are retrospective, single center observational studies, and therefore prone to a degree of selection bias. The retrospective nature along with the relatively long time period these studies cover may carry unknown variables affecting outcomes that remain unaccounted for in the final analysis in all of these studies. Furthermore, as the population size for elderly especially octogenarians remains relatively small when compared to the total volume of most surgical units therefore size of the study populations in most studies remains small. Last but not the least, majority of the studies report short-term or early outcomes and there is a need for further studies with longer follow-up. 

## CONCLUSION

The elderly are the fastest growing segment of the CABG patient population. These patients often have concomitant diseases, such as renal insufficiency, chronic obstructive pulmonary disease, peripheral vascular disease, prostatic enlargement complicated by urinary retention, and degenerative cerebral disease. The overall reduction in the operative mortality and morbidity after CABG due to recent technological advances in surgical and anesthetic techniques and improvements in postoperative care has made CABG a valid option of treatment for this group of patients. The decision for surgery is complex in this group of patients and one must take into account several elements, such as the lack of synchronism between physiological age and chronological age, the quality of life, and the risk– benefit ratio. Furthermore, the risk of death from a cardiac operation in elderly patients can be reduced to that of younger patients with consistent and careful application of modern techniques and clinical practices. OPCAB, due to its less invasive nature compared to conventional CABG, appears an attractive option to reduce procedure specific mortality and morbidity in the elderly. However, current observational studies with small numbers, examining the impact of avoiding CPB on early mortality and morbidity in the elderly, have yielded variable results. It is expected that in the coming years adequately powered studies, with randomized design and long-term follow-up, will provide more concrete evidence to verify the safety and efficacy of OPCAB in the elderly.

## Figures and Tables

**Table 1.** Studies Comparing Outcomes of On-pump and Off-pump Coronary Artery Bypass Grafting in Elderly

Author, Reference (Year)	No. of Patients OPCAB/CPB	Type of Study	Level of Evidence	Definition of Elderly (Years)	Exclusion Criteria*
Saleh [66] (2011)	156/187	Retrospective†	2b	>80	3, 5
LaPar [67] (2011)	404/1589	Retrospective	2b	>80	3, 5
Tugtekin [71] (2007)	107/237	Retrospective	2b	>80	3
Meco [72] (2007)	78/41	Retrospective†	2b	>75	3
D'Alfonso [81] (2004)	73/41	Retrospective	2b	>80	ND
Deuse [87] (2003)	53/66	Retrospective	2b	>75	ND
Meharwal [88] (2002)	186/389	Retrospective	2b	>70	3, 4
Demaria [89] (2002)	62/63	Retrospective	2b	>80	ND
Hoff [90] (2002)	59/69	Retrospective	2b	>80	1, 2
Ascione [91] (2002)	219/771	Retrospective	2b	>70	ND
Hirose [93] (2001)	104/74	Retrospective	2b	>75	ND
Al-Ruzzeh [94] (2001)	56/87	Retrospective	2b	>75	2-4
Ricci [95] (2000)	97/172	Retrospective	2b	>80	Nil
Koutlas [96] (2000)	53/220	Retrospective	2b	>75	3, 5
Boyd [97] (1999)	30/70	Retrospective	2b	>70	ND

* Exclusion criteria: 1, emergency operation; 2, minimally invasive direct coronary artery bypass grafting; 3, coronary grafting in combination with other cardiac surgical procedures; 4, <2 grafts; 5, repeat surgery; 6, catastrophic stage: unstable hemodynamic status or severe ischemia that could not be stabilized with inotropes or intra-aortic balloon pump.

ND = not defined

† Propensity matched analysis

**Table 2.** Outcomes of On-pump and Off-pump Coronary Artery Bypass Grafting in Elderly

Author, Reference (year)	No. of Patients OPCAB/CPB	Mortality(%) OPCAB/CPB	Stroke(%) OPCAB/CPB	AF(%) OPCAB/CPB	RF(%) OPCAB/CPB	LOS(days) OPCAB/CPB
Saleh[66] (2011)	156/187	4.7/6.5	1.9/5.4	28.2/43.9	13.5/15.5	10/9
LaPar[67] (2011)	404/1589	5.9/5.1	1.7/2.6	21.5/28.4	6.2/8.1	12.3/12.2
Tugtekin [71] (2007)	107/237	3.7/5.1	0/1.3	23.3/23.3	2.8/2.1	NR
Meco [72] (2007)	78/41	1.3/12.2*	0/4.9	21.8/26.8	0/12.2	10.5/23.3*
D'Alfonso [81] (2004)	73/41	6.8/14.6*	1.4/2.4	4.1/14.7	2.4/2.6	NR
Deuse [87] (2003)	53/66	7.5/9.1	0/0	NR	NR	9.6/9.7
Meharwal [88] (2002)	186/389	2.2/4.6	0/0.5	10.2/18.5	1.1/2.1	5/8*
Demaria [89] (2002)	62/63	4.8/15.9*	0/6.3*	54.8/61.5	19.8/14.5	9/9.6
Hoff [90] (2002)	59/69	0/4.7	0/7.1*	23.3/30.8	0/1.8	6.3/11.5*
Ascione [91] (2002)	219/771	1.4/2.1	0.46/1	12.3/14	0.9/1.9	8.7/8.7
Hirose [93] (2001)	104/74	1.9/0	1/8.1*	NR	1.9/1.4	13.8/20
Al-Ruzzeh [94] (2001)	56/87	0/11*	0/5*	29/41	0/7*	9.7/10.7
Ricci [95] (2001)	97/172	5.2/10.3	0/9.3*	NR	4.1/4.1	9.1/10.8
Koutlas [96] (2000)	53/220	0/7.6*	2.2/2.3	26/26	0/3.5	4.4/8.4*
Boyd [97] (1999)	30/70	0/1.7	0/6.7	10/28.3*	NR	6.3/7.7*

AF = atrial fibrillation; CPB = cardiopulmonary bypass; LOS = length of stay; NA = not reported; RF = renal failure; OPCAB = off-pump coronary artery bypass

* p < 0.05

**Table 3.** Outcomes of Off-pump and On-pump Coronary Artery Bypass Grafting in Octogenarians at Harefield Hospital (January 2001-December 2010)

Variable	OPCAB	ONCAB	*p* value
Number of patients	217	73	<0.001
Age (range, years)	80-89	80-88	
Age (mean, years)	82.04	81.89	NS
Females	73 (33.6)	17 (23.3)	NS
Logistic EuroSCORE	9.5	8.8	NS
Mortality	17 (7.8)	5 (6.8)	NS
AF/SVT	86 (39.6)	31 (42.5)	NS
Renal failure	46 (21.2)	15 (20.5)	NS
Inotropes	61 (28.1)	37 (50.7)	<0.001
Stroke/TIA	1/1 (0.9)	1/1 (2.7)	0.04
Chest infection	35 (16.1)	13 (17.8)	NS
GI complications	14 (6.5)	7 (9.6)	0.04
Re-exploration for bleeding	9 (4.1)	14 (19.2)	<0.001
Sternal wound infection			
Superficial	14 (6.5)	2 (1.4)	0.02
Deep	5 (2.3)	2 (1.4)	NS
Blood product usage	58 (26.7)	49 (67.1)	<0.001
ITU LOS (hours)	27.2	53.9	<0.001
Hospital LOS (days)	15.97	17.25	0.03

AF = atrial fibrillation; GI = gastrointestinal; ITU = intensive therapy unit; LOS = length of stay; NS = not significant; ONCAB = on-pump coronary artery bypass; OPCAB = off-pump coronary artery bypass; SVT = supraventricular tachycardia; TIA = transient ischemic attack

## References

[1] Ivanov J, Weisel RD, David TE (1998). Fifteen?year trends in risk severity and operative mortality in elderly patients undergoing coronary artery bypass graft surgery. Circulation.

[2] Natarajan A, Samadian S, Clark S (2007). Coronary artery bypass surgery in elderly people. Postgrad Med J.

[3] Moshkovitz Y, Paz Y, Shabtai E (1997). Predictors of early and overall outcome in coronary artery bypass without cardiopulmonary bypass. Eur J Cardiothorac Surg.

[4] Loop FD, Lytle BW, Cosgrove DM (1988). Coronary artery bypass graft surgery in the elderly. Indications and outcome. Cleve Clin J Med.

[5] Lakatta EG (1990). Changes in cardiovascular function with aging. Eur Heart J.

[6] Loran DB, Zwischenberger JB (2004). Thoracic surgery in the elderly. J Am Coll Surg.

[7] Folkow B, Svanborg A (1993). Physiology of cardiovascular aging. Physiol Rev.

[8] Lakatta EG (1999). Cardiovascular aging research: the next horizons. J Am Geriatr Soc.

[9] Gajraj RJ, Doi M, Mantzaridis H (1999). Comparison of bispectral EEG analysis and auditory evoked potentials for monitoring depth of anaesthesia during propofol anaesthesia. Br J Anaesth.

[10] Lakatta EG (1993). Cardiovascular regulatory mechanisms in advanced age. Physiol Rev.

[11] Olivetti G, Melissari M, Capasso JM (1991). Cardiomyopathy of the aging human heart. Myocyte loss and reactive cellular hypertrophy. Circ Res.

[12] Lakatta E (1994). Aging effects on the vasculature in health: risk factors for cardiovascular disease. Am J Geriatr Cardiol.

[13] Priebe HJ (2000). The aged cardiovascular risk patient. Br J Anaesth.

[14] Czernin J, Müller P, Chan S (1993). Influence of age and hemodynamics on myocardial blood flow and flow reserve. Circulation.

[15] Egashira K, Inou T, Hirooka Y (1993). Effects of age on endothelium-dependent vasodilation of resistance coronary artery by acetylcholine in humans. Circulation.

[16] Amrani M, Goodwin AT, Gray CC, Yacoub MH (1996). Aging is associated with reduced basal and stimulated release of nitric oxide by the coronary endothelium. Acta Physiol Scand.

[17] Goodwin AT, Amrani M, Marchbank AJ (1999). Coronary vasoconstriction to endothelin-1 increases with age before and after ischaemia and reperfusion. Cardiovasc Res.

[18] Miyauchi T, Yanagisawa M, Iida K (1992). Age- and sex-related variation of plasma endothelin-1 concentration in normal and hypertensive subjects. Am Heart J.

[19] Barakat K, Wilkinson P, Deaner A (1999). How should age affect management of acute myocardial infarction? A prospective cohort study. Lancet.

[20] Detsky AS, Abrams HB, McLaughlin JR (1986). Predicting cardiac complications in patients undergoing non-cardiac surgery. J Gen Intern Med.

[21] Alfonso F, Azcona L, Perez-Vizcayno MJ (1999). Initial results and long-term clinical and angiographic implications of coronary stenting in elderly patients. Am J Cardiol.

[22] Paul SD, O'Gara PT, Mahjoub ZA (1996). Geriatric patients with acute myocardial infarction: cardiac risk factor profiles, presentations, thrombolysis, coronary interventions and prognosis. Am Heart J.

[23] Cartier R (2009). Off-pump coronary artery revascularization in octogenarians: is it better?. Curr Opin Cardiol.

[24] Centers for Disease Control and Prevention (2003). Trends in aging: United States and worldwide. Morb Mortal Wkly Rep.

[25] Granger CB, Goldberg RJ, Dabbous O, Global Registry of Acute Coronary Events Investigators (2003). Predictors of hospital mortality in the Global Registry of Acute Coronary Events. Arch Intern Med.

[26] Avezum A, Makdisse M, Spencer F, GRACE Investigators (2005). Impact of age on management and outcome of acute coronary syndrome: observations from the Global Registry of Acute Coronary Events (GRACE). Am Heart J.

[27] Lee PY, Alexander KP, Hammill BG (2001). Representation of elderly persons and women in published randomized trials of acute coronary syndromes. JAMA.

[28] TIME Investigators (2001). Trial of invasive versus medical therapy in elderly patients with chronic symptomatic coronary-artery disease (TIME): a randomised trial. Lancet.

[29] Graham MM, Ghali WA, Faris PD (2002). Alberta Provincial Project for Outcomes Assessment in Coronary Heart Disease (APPROACH) Investigators. Survival after coronary revascularization in elderly. Circulation.

[30] Alexander KP, Roe MT, Chen AY (2005). Evolution in cardiovascular care for elderly patients with non-ST-segment elevation acute coronary syndromes: results from the CRUSADE National Quality Improvement Initiative. J Am Coll Cardiol.

[31] Peterson ED, Alexander KP, Malenka DJ, American Heart Association Chronic CAD Working Group (2004). American Heart Association Chronic CAD working group. Multicenter experience in revascularization of very elderly patients. Am Heart J.

[32] Batchelor WB, Anstrom KJ, Muhlbaier LH (2000). Contemporary outcome trends in the elderly undergoing percutaneous coronary interventions: results in 7,472 octogenarians. National Cardiovascular Network Collaboration. J Am Coll Cardiol.

[33] De Gregorio? J, Kobayashi Y, Albiero R (1998). Coronary artery stenting in the elderly: short-term outcome and long-term angiographic and clinical follow-up. J Am Coll Cardiol.

[34] Kowalchuk? CJ, Siu SC, Lewis SM (1990). Coronary artery disease in the octogenarian: angiographic spectrum and suitability for revascularization. Am J Cardiol.

[35] Klein? LW, Block P, Brindis RG (2002). ACC-NCDR registry. Percutaneous coronary interventions in octogenarians in the American College of Cardiology-National Cardiovascular Data Registry. Development of a nomogram predictive of in-hospital mortality. J Am Coll Cardiol.

[36] Hermiller JB, Nikolsky E, Lansky AJ (2011). Clinical and angiographic outcomes of elderly patients treated with everolimus-eluting versus paclitaxel-eluting stents: three-year results from the SPIRIT III randomised trial. EuroIntervention.

[37] Hedeshian M H, Namour N, Dziadik E (2002). Does increasing age have a negative impact on six-month functional outcome after coronary artery bypass?. Surgery.

[38] Zingone B, Gatti G, Rauber E (2009). Early and late outcomes of cardiac surgery in octogenerians. Ann Thorac Surg.

[39] Maganti M, Rao V, Brister S, Ivanov J (2009). Decreasing mortality for coronary artery bypass surgery in octogenarians. Can J Cardiol.

[40] Hlatky MA, Boothroyd DB, Bravata DM (2009). Coronary artery bypass surgery compared with percutaneous coronary interventions for multivessel disease: a collaborative analysis of individual patient data from ten randomised trials. Lancet.

[41] Mortasawi A, Arnrich B, Walter J (2004). Impact of age on the results of coronary artery bypass grafting. Asian Cardiovasc Thorac Ann.

[42] Wilson M F, Baig M K, Ashraf H (2005). Quality of life in octagenarians after coronary artery bypass grafting. Am J Cardiol.

[43] Dacey LJ, Likosky DS, Ryan TJ, Northern New England Cardiovascular Disease Study Group (2007). Long-term survival after surgery versus percutaneous intervention in octogenarians with multivessel coronary disease. Ann Thorac Surg.

[44] Artinian N T, Duggan C, Miller P (1993). Age differences in patient recovery patterns following coronary artery bypass surgery. Am J Crit Care.

[45] Rumsfeld J S, Magid D J, O'Brien M (2001). Changes in health-related quality of life following coronary artery bypass graft surgery. Ann Thorac Surg.

[46] Raja SG, Dreyfus GD (2006). Impact of off-pump coronary artery bypass surgery on post-operative pulmonary dysfunction: current best available evidence. Ann Card Anaesth.

[47] Raja SG, Berg GA (2007). Impact of off-pump coronary artery bypass surgery on systemic inflammation: current best available evidence. J Card Surg.

[48] Marti L, Cervera C, Filella X (2007). Cytokine-release patterns in elderly patients with systemic inflammatory response syndrome. Gerontology.

[49] Deng M C, Dasch B, Erren M (1996). Impact of left ventricular dysfunction on cytokines, hemodynamics, and outcome in bypass grafting. Ann Thorac Surg.

[50] Raja SG, Berg GA (2007). Outcomes of off-pump coronary artery bypass surgery: current best available evidence. Indian Heart J.

[51] Sedrakyan A, Wu AW, Parashar A (2006). Off-pump surgery is associated with reduced occurrence of stroke and other morbidity as compared with traditional coronary artery bypass grafting: a meta-analysis of systematically reviewed trials. Stroke.

[52] Parolari A, Alamanni F, Cannata A (2003). Off-pump versus on-pump coronary artery bypass: meta-analysis of currently available randomized trials. Ann Thorac Surg.

[53] Reston JT, Tregear SJ, Turkelson CM (2003). Meta-analysis of short-term and mid-term outcomes following off-pump coronary artery bypass grafting. Ann Thorac Surg.

[54] Raja SG, Dreyfus GD (2006). Impact of off-pump coronary artery bypass surgery on postoperative renal dysfunction: current best available evidence. Nephrology (Carlton).

[55] Raja SG, Dreyfus GD (2006). Impact of off-pump coronary artery bypass surgery on postoperative bleeding: current best available evidence. J Card Surg.

[56] Raja SG, Dreyfus GD (2008). Current status of off-pump coronary artery bypass surgery. Asian Cardiovasc Thorac Ann.

[57] Mangano CM, Diamondstone LS, Ramsay JG (1998). Renal dysfunction after myocardial revascularization: risk factors, adverse outcomes, and hospital resource utilization. Ann Intern Med.

[58] Amar D, Zhang H, Leung DH (2002). Older age is the strongest predictor of postoperative atrial fibrillation. Anesthesiology.

[59] Al-Ruzzeh S, George S, Bustami M (2006). Effect of off-pump coronary artery bypass surgery on clinical, angiographic, neurocognitive and quality of life outcomes: randomised controlled trial. Br Med J.

[60] Stamou SC, Dangas G, Dullum MK (2000). Beating heart surgery in octogenarians: perioperative outcome and comparison with younger age groups. Ann Thorac Surg.

[61] Guru V, Glasgow KW, Fremes SE (2007). The real-world outcomes of off-pump coronary artery bypass surgery in a public healthcare system. Can J Cardiol.

[62] Athanasiou T, Al-Ruzzeh S, Kumar P (2004). Off-pump myocardial revascularization is associated with less incidence of stroke in elderly patients. Ann Thorac Surg.

[63] Guerra M, Carlos Mota J (2008). Adult cardiac surgery: impact on age group differences in preoperative risk factors and early mortality. Rev Port Cir Cardiotorac Vasc.

[64] Scott BH, Seifert FC, Grimson R, Glass PS (2005). Octogenarians undergoing coronary artery bypass graft surgery: resource utilization, postoperative mortality, and morbidity. J Cardiothorac Vasc Anesth.

[65] Panesar SS, Athanasiou T, Nair S (2006). Early outcomes in the elderly: a meta-analysis of 4921 patients undergoing coronary artery bypass grafting – comparison between off-pump and on-pump techniques. Heart.

[66] Saleh HZ, Shaw M, Fabri BM (2011). Does avoidance of cardiopulmonary bypass confer any benefits in octogenarians undergoing coronary surgery?. Interact Cardiovasc Thorac Surg.

[67] LaPar DJ, Bhamidipati CM, Reece TB (2011). Is off-pump coronary artery bypass grafting superior to conventional bypass in octogenarians?. J Thorac Cardiovasc Surg.

[68] Serrão M, Graça F, Rodrigues R (2010). Coronary artery bypass grafting in octogenarians: long-term results. Rev Port Cardiol.

[69] Pinto E, Silva AM, Campagnucci VP, Pereira WL (2008). Off-pump myocardial revascularization in the elderly: analysis of morbidity and mortality. Rev Bras Cir Cardiovasc.

[70] Jensen B, Rasmussen LS, Steinbrüchel DA (2008). Cognitive outcomes in elderly high-risk patients 1 year after off-pump versus on-pump coronary artery bypass grafting. A randomized trial. Eur J Cardiothorac Surg.

[71] Tugtekin S, Kappert U, Alexiou K (2007). Coronary artery bypass grafting in octogenarians--outcome with and without extracorporeal circulation. Thorac Cardiovasc Surg.

[72] Meco M, Biraghi T, Panisi P (2007). Aortocoronary bypass grafting
in high-risk patients over 75 years. Propensity score analysis of on
versus off-pump, early and midterm results. J Cardiovasc Surg (Torino).

[73] Jensen B, Hughes P, Rasmussen LS (2006). Health-related quality of life following off-pump versus on-pump coronary artery bypass grafting in elderly moderate to high-risk patients: a randomized trial. Eur J Cardiothorac Surg.

[74] Jensen BØ, Hughes P, Rasmussen LS (2006). Cognitive outcomes in elderly high-risk patients after off-pump versus conventional coronary artery bypass grafting: a randomized trial. Circulation.

[75] Kitamura H, Okabayashi H, Hanyu M (2005). Early and late results and problems of coronary artery bypass grafting in patients over 80-year-old. Kyobu Geka.

[76] Tomita S, Watanabe G (2005). Performing off-pump coronary artery bypass grafting in elderly patients safely. Kyobu Geka.

[77] Sugimoto T, Yamamoto K, Tanaka S (2005). Off-pump versus conventional coronary artery bypass grafting in octogenarians. Kyobu Geka.

[78] Nakamura Y, Nakano K, Nakatani H (2004). Hospital and mid-term outcomes in elderly patients under-going off-pump coronary artery bypass grafting--comparison with younger patients--. Circ J.

[79] Xue S, Xie B, Liu S (2004). Off-pump coronary artery bypass grafting in patients over the age of seventy. Zhonghua Wai Ke Za Zhi.

[80] Tanaka H, Narisawa T, Masuda M (2004). Coronary artery bypass in patients 80 years and older: comparison with a younger age group. Ann Thorac Cardiovasc Surg.

[81] D'Alfonso A, Mariani M A (2004). Off-pump coronary surgery improves in-hospital and early outcomes in octogenarians. Ital Heart J.

[82] Carrier M, Perrault LP, Jeanmart H (2003). Randomized trial comparing off-pump to on-pump coronary artery bypass grafting in high-risk patients. Heart Surg Forum.

[83] Matsumoto Y, Endo M, Kasashima F (2003). Off-pump coronary artery bypass grafting for octogenarians with acute coronary syndrome. Kyobu Geka.

[84] Beauford RB, Goldstein DJ, Sardari FF (2003). Multivessel off-pump revascularization in octogenarians: early and midterm outcomes. Ann Thorac Surg.

[85] Lin CY, Hong GJ, Lee KC (2003). Off-pump technique in coronary artery bypass grafting in elderly patients. Aust N Z J Surg.

[86] Shimokawa T, Minato N, Yamada N (2003). Off-pump coronary artery bypass grafting in octogenarians. Jpn J Thorac Cardiovasc Surg.

[87] Deuse T, Detter C, Samuel V (2003). Early and midterm results after coronary artery bypass grafting with and without cardiopulmonary bypass: which patient population benefits the most?. Heart Surg Forum.

[88] Meharwal Z S, Trehan N (2002). Off-pump coronary artery surgery in the elderly. Asian Cardiovasc Thorac Ann.

[89] Demaria RG, Carrier M, Fortier S (2002). Reduced mortality and strokes with off-pump coronary artery bypass grafting surgery in octogenarians. Circulation.

[90] Hoff SJ, Ball SK, Coltharp WH (2002). Coronary artery bypass in patients 80 years and over: is off-pump the operation of choice?. Ann Thorac Surg.

[91] Ascione R, Rees K, Santo K (2002). Coronary artery bypass grafting in patients over 70 years old: the influence of age and surgical technique on early and mid-term clinical outcomes. Eur J Cardiothorac Surg.

[92] Ricci M, Karamanoukian HL, Dancona G (2001). On-pump and off-pump coronary artery bypass grafting in the elderly: predictors of adverse outcome. J Card Surg.

[93] Hirose H, Amano A, Takahashi A (2001). Off-pump coronary artery bypass grafting for elderly patients. Ann Thorac Surg.

[94] Al?Ruzzeh S, George S, Yacoub M (2001). The clinical outcome of off-pump coronary artery bypass surgery in the elderly patients. Eur J Cardiothorac Surg.

[95] Ricci M, Karamanoukian HL, Abraham R (2000). Stroke in octogenarians undergoing coronary artery surgery with and without cardiopulmonary bypass. Ann Thorac Surg.

[96] Koutlas TC, Elbeery JR, Williams JM (2000). Myocardial revascularization in the elderly using beating heart coronary artery bypass surgery. Ann Thorac Surg.

[97] Boyd WD, Desai ND, Del Rizzo DF (1999). Off-pump surgery decreases postoperative complications and resource utilization in the elderly. Ann Thorac Surg.

[98] Johnson WM, Smith M, Woods SE (2005). Cardiac surgery in octogenarians: does age alone influence outcomes?. Arch Surg.

[99] Furlan AJ, Sila CA, Chimowitz MI (1992). Neurologic complications related to cardiac surgery. Neurol Clin.

[100] Stamou SC, Dangas G, Dullum MK (2000). Beating heart surgery in octogenarians: perioperative outcome and comparison with younger age groups. Ann Thorac Surg.

[101] Puskas JD, Winston D, Wright CE (2000). Stroke after coronary artery operations: incidence, correlates, outcome, and cost. Ann Thorac Surg.

[102] McKhann G, Goldsborough M, Borowicz L (1997). Predictors of stroke risk in coronary artery bypass patients. Ann Thorac Surg.

[103] Ascione R, Reeves BC, Chamberlain MH (2002). Predictors of stroke in the modern era of coronary artery bypass grafting: a case control study. Ann Thorac Surg.

[104] Hammon JW, Stump DA, Kon ND (1997). Risk factors and solutions for the development of neurobehavioral changes after coronary artery bypass grafting. Ann Thorac Surg.

[105] Athanasiou T, Aziz O, Mangoush O (2004). Do off-pump techniques reduce the incidence of postoperative atrial fibrillation in elderly patients undergoing coronary artery bypass grafting?. Ann Thorac Surg.

[106] Fearn SJ, Pole R, Wesnes K (2001). Cerebral injury during cardiopulmonary bypass: emboli impair memory. J Thorac Cardiovasc Surg.

[107] Diegeler A, Hirsch R, Schneider F (2000). Neuromonitoring and neurocognitive outcome in off-pump versus conventional coronary bypass operation. Ann Thorac Surg.

[108] Zamvar V, Williams D, Hall J (2002). Assessment of neurocognitive impairment after off-pump and on-pump techniques for coronary artery bypass graft surgery: prospective randomised controlled trial. BMJ.

[109] Lee JD, Lee SJ, Tsushima WT (2003). Benefits of off-pump bypass on neurologic and clinical morbidity: a prospective randomized trial. Ann Thorac Surg.

[110] Selnes OA, McKhann GM (2005). Neurocognitive complications after coronary artery bypass surgery. Ann Neurol.

[111] van Dijk D, Jansen EW, Hijman R, Octopus Study Group (2002). Cognitive outcome after off-pump and on-pump coronary artery bypass graft surgery: a randomized trial. JAMA.

[112] van Dijk D, Spoor M, Hijman R, Octopus Study Group (2007). Cognitive and cardiac outcomes 5 years after off-pump vs on-pump coronary artery bypass graft surgery. JAMA.

[113] Rasmussen LS, Larsen K, Houx P (2001). The assessment of postoperative cognitive function. Acta Anaesthesiol Scand.

[114] Browne SM, Halligan PW, Wade DT (1999). Cognitive performance after cardiac operation: implications of regression toward the mean. J Thorac Cardiovasc Surg.

[115] Newman MF, Kirchner JL, Phillips-Bute B, The Neurological Outcome Research Group and the Cardiothoracic Anesthesiology Research Endeavors Investigators (2001). Longitudinal assessment of neurocognitive function after coronary-artery bypass surgery. N Eng J Med.

[116] Nierich AP, Diephuis J, Jansen EW (2000). Heart displacement during off-pump CABG: how well is it tolerated?. Ann Thorac Surg.

[117] Mariscalco G, Engström KG (2009). Postoperative atrial fibrillation is associated with late mortality after coronary surgery, but not after valvular surgery. Ann Thorac Surg.

[118] Maisel WH, Rawn JD, Stevenson WG (2001). Atrial fibrillation after cardiac surgery. Ann Intern Med.

[119] Mathew JP, Fontes ML, Tudor IC (2004). A multicenter risk index for atrial fibrillation after cardiac surgery. JAMA.

[120] Athanasiou T, Aziz O, Mangoush O (2004). Does off-pump coronary artery bypass reduce the incidence of postoperative atrial fibrillation? A question revisited. Eur J Cardiothorac Surg.

[121] Maitra G, Ahmed A, Rudra A (2009). Renal dysfunction after off-pump coronary artery bypass surgery- risk factors and preventive strategies. Indian J Anaesth.

[122] Weerasinghe A, Athanasiou T, Al-Ruzzeh S (2005). Functional renal outcome in on-pump and off-pump coronary revascularization: a propensity-based analysis. Ann Thorac Surg.

[123] Nigwekar SU, Kandula P, Hix JK (2009). Off-pump coronary artery bypass surgery and acute kidney injury: a meta-analysis of randomized and observational studies. Am J Kidney Dis.

[124] Rothenhausler HB, Grieser B, Nollert G (2005). Psychiatric and psychosocial outcome of cardiac surgery with cardiopulmonary bypass: a prospective 12-month follow-up study. Gen Hosp Psychiatry.

[125] Newman MF, Grocott HP, Mathew JP (2001). Report of the substudy assessing the impact of neurocognitive function on quality of life 5 years after cardiac surgery. Stroke.

[126] Mortensen OS, Madsen JK, Haghfelt T, DANAMI study group (2000). Health related quality of life after conservative or invasive treatment of inducible postinfarction ischaemia. Heart.

[127] Duits AA, Boeke S, Taams MA (1997). Prediction of quality of life after coronary artery bypass graft surgery: a review and evaluation of multiple, recent studies. Psychosom Med.

